# Metastatic adenocarcinoma of the colon presenting as a monarthritis of the hip in a young patient

**DOI:** 10.1186/1477-7819-4-95

**Published:** 2006-12-14

**Authors:** Neil Ruparelia, Hootan Ahmadi, Carlos Cobiella

**Affiliations:** 1 Department of Orthopaedic Surgery, University College Hospital, 235 Euston Road, London NW1 2BU, UK

## Abstract

**Background:**

Malignant arthritis is a rare manifestation of metastatic disease. We describe the case of a previously well 28 year old man in whom hip pain was the presenting symptom of disease. We describe the case and discuss the aetiology of colorectal cancer in young patients. We then review the literature and discuss the investigation and management of malignant joint arthritis.

**Case presentation:**

We present the case of a 28 year old man who presented to the emergency department with an acute monoarthritis of the hip. He had an unremarkable past medical history and was systemically well. A diagnosis of malignant joint effusion was reached after a heightened index of clinical suspicion, magnetic resonance imaging and cytological evaluation of the synovial fluid. Computed tomography and bone scan confirmed widespread metastatic disease from a primary colonic adenocarcinoma. The patient tolerated three cycles of oxaliplatin and capecitabine but died 4 months after presentation.

**Conclusion:**

The metastatic spread of cancer to the joint and the synovium is one of the rarest manifestations of malignant disease and has not been previously reported as the presenting symptom of disease. The diagnosis is a difficult one to reach and is associated with a poor prognosis. This case illustrates the importance of thorough investigation in reaching this diagnosis and entertaining the possibility in individuals who do not respond to conventional management of acute monoarthritis, even in young patients and individuals who do not display any other symptoms of disease.

## Background

Colorectal cancer usually presents in patients in their 6^th ^to 8^th ^decade or in individuals in whom there is a genetic predisposition to develop cancer. The usual presenting symptoms are commonly those of altered bowel habit, abdominal pain, bleeding per anum, general malaise or a microcytic anaemia of unexplained aetiology.

We present the unusual case of a young patient with no apparent risk factors or genetic predisposition for colorectal cancer who presented with an acute monoarthritis of the hip as the first symptom of his disease.

## Case presentation

A 28 year old male student of Nepalese origin living in the United Kingdom for 3 years presented to the Accident and Emergency department with a 3 week history of increasing right hip pain, with associated fevers but no other symptoms and no history of trauma. 5 years previously, he had been treated for pulmonary tuberculosis with a 6 month course of chemotherapy. There was no positive family history. The remaining history was unremarkable.

**Figure 1:**
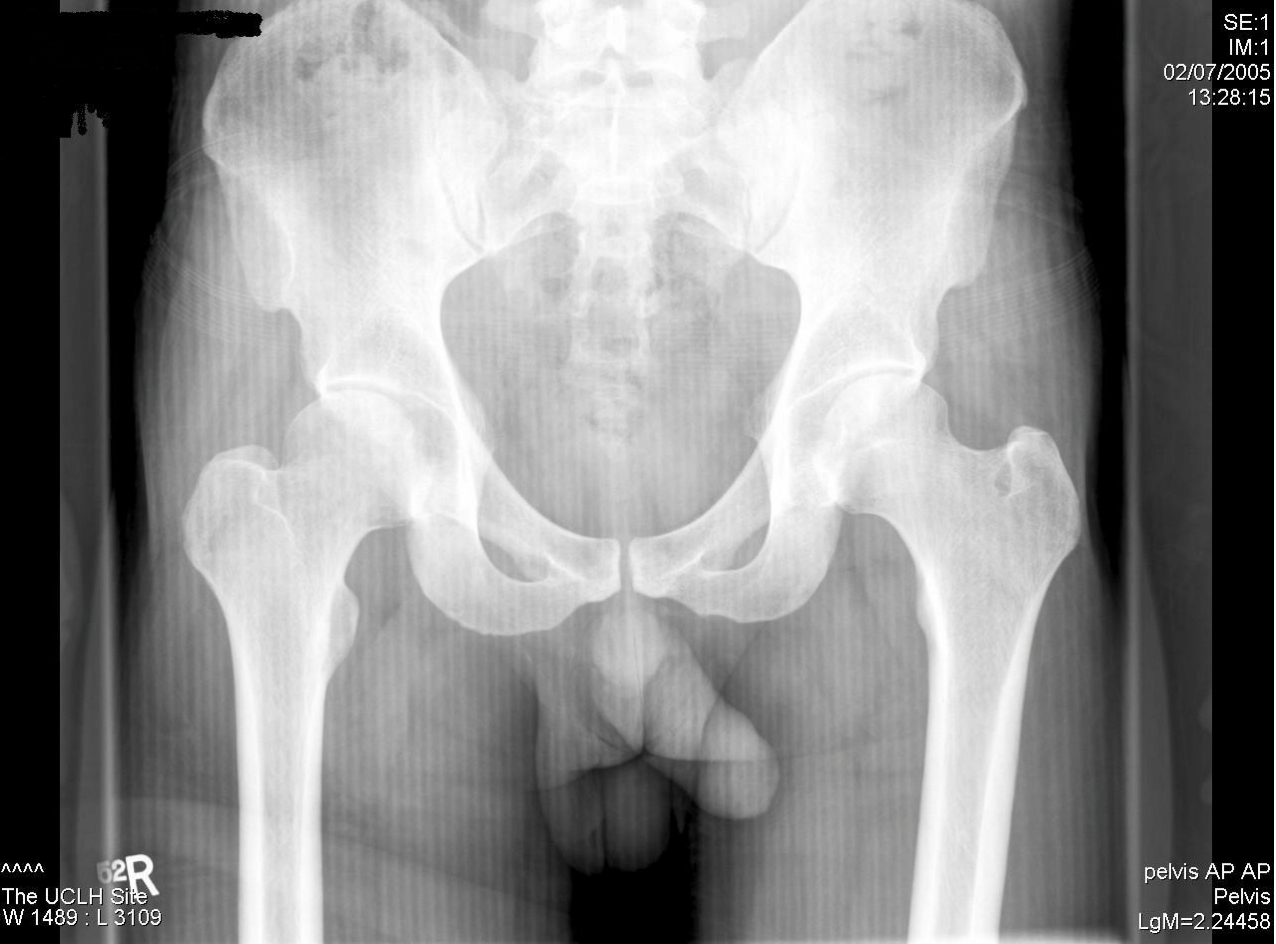
X-ray of the pelvis showing no positive findings.

On admission he was partially weight bearing on the affected side. On inspection there were no signs of inflammation. Flexion was limited to100 degrees; extension to 20 degrees; internal and external rotation were reduced to 10 degrees with normal adduction and abduction. There were no further positive findings on the remainder of physical examination or X-ray (figure 1).

Blood results on admission were haemoglobin 12.5 g/dl (12.5–18.0 g/dl), white cell count 6.47 (4.0–11.0 × 10^9^/l), Platelets 378 (150–400 × 10^9^/l), C reactive protein (CRP) 205 (<5 mg/l), Erythrocyte sedimentation rate (ESR) 96 (2–12 mm/1^st ^hour). His biochemistry revealed an alkaline phosphatase (ALP) 342 (30–300 iu/L), bilirubin 15 (3–17 μmol/L), alanine aminotransferase (ALT) 45 (3–35 iu/L), aspartate transaminase (AST) 42 (3–35 iu/L), corrected calcium 2.82 (2.12–2.65 mmol/L), lactate dehydrogenase (LDH) 782 (iu/L). Renal function was normal.

**Figure 2:**
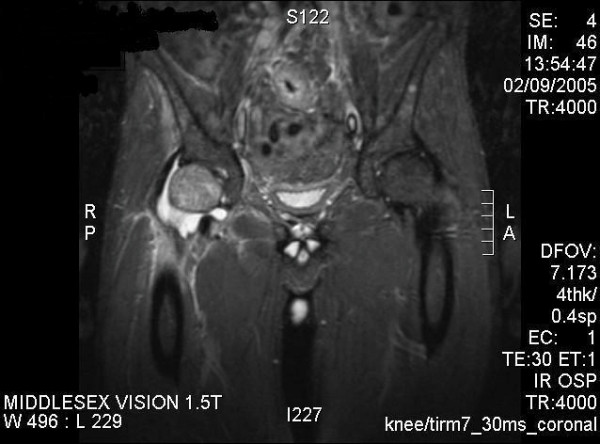
MRI of the pelvis showing effusion with soft tissue swelling and synovial thickening.

An Ultrasound of the hip revealed a moderate right sided joint effusion. Magnetic resonance imaging (MRI) of the pelvis and hips (figure 2) confirmed the effusion with marked soft tissue swelling and synovial thickening. There was oedema in the proximal femur.

Our working diagnosis was septic arthritis, with tuberculosis of the hip as a differential, in view of his past medical history. He was thus managed accordingly with a washout and commenced on chemotherapy. Microbiology of the synovial fluid revealed no organisms; auramine tests were repeatedly negative, as was syphilis serology, Human Immunodeficiency Virus (HIV), Hepatitis and melioidosis.

There was little clinical response to treatment and inflammatory markers 2 weeks after admission were elevated at a CRP of 130 and ESR of 114.

He then developed an episode of diarrhoea and vomiting as an inpatient. Initial investigations for amoeba, cysts, ova, parasites were all negative. Colonoscopy was undertaken and revealed a stricture in the sigmoid colon. Multiple biopsies revealed a moderately to poorly differentiated adenocarcinoma on histological examination. An immunohistochemical panel showed the tissue to be cytokeratin 20 (CK-20) positive, cytokeratin 7 (CK-7) negative and villin negative, thus in keeping with a colorectal primary tumour. Carcinoembryonic antigen (CEA) was measured at 100.7. Genetic screening and microsatellite instability (MSI) testing on the tumour were negative.

The synovial fluid was re-examined for cytology following the histological diagnosis and this confirmed a malignant joint effusion.

**Figure 3:**
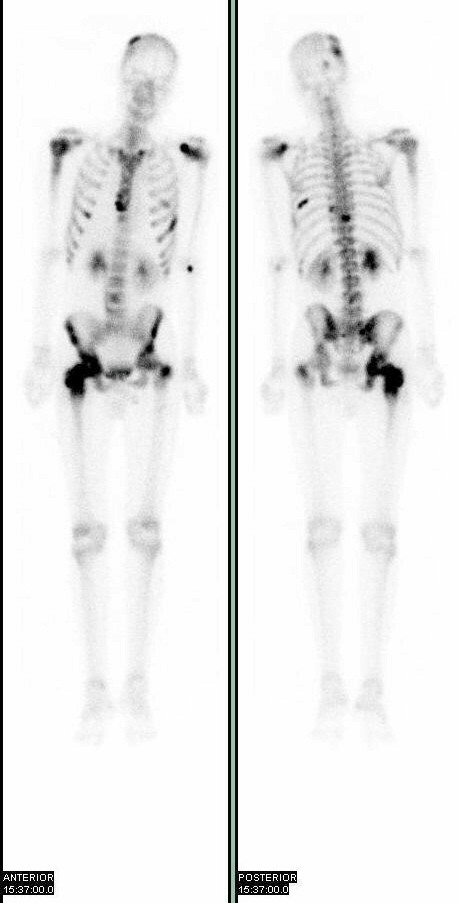
Bone Scan showing high uptake in skull vault, spine, ribs, pelvis, both femur and sternum suggestive of widespread metastatic disease.

A computerised tomogram (CT) revealed multiple para-aortic lymph nodes, with no evidence of metastases other solid organs. A bone scan (figure 3) revealed high uptake areas in the skull vault, spine, ribs, pelvis, proximal ends of both femurs and sternum; all in keeping with widespread metastatic disease.

In view of the advanced nature of the adenocarcinoma the primary tumour was not resected and he was commenced on Oxaliplatin 130 mg/m^2 ^(day one of cycle) and Capecitabine 1.25 g/m^2 ^(twice daily for fourteen days) for a planned eight cycles (each cycle of twenty-one days). He responded well initially with an improvement symptomatically after 2 cycles of chemotherapy, and objectively on computerised tomogram (CT) of his abdomen and pelvis with reduction in the size and number of significant lymph nodes. However was unable to tolerate more than 3 cycles and died 4 months after his initial presentation.

## Discussion

Adenocarcinoma of the colon in young patients is rare with only 2% of diagnosed colonic cancers in patients 40 years or younger [1]. Risk factors include inflammatory bowel disease and low fibre diet but the majority in this age group are hereditary being attributable to two recognised syndromes.

The first is familial adenomatous polyposis (FAP); the underlying cause is a gene mutation adenomatous polyposis coli (APC) gene on chromosome 5. This usually has a clear phenotype, characterised by numerous adenomatous polyps in the large bowel by the third decade of life and invariably develop into cancers.

The second is hereditary non-polyposis colorectal cancer (HNPCC) syndrome. Here the colorectal cancer also arises from adenomas, but the degree of polyposis is less marked than in FAP. This syndrome has a heterogeneous spectrum, and the phenotype is more difficult to define than in FAP [2]. It is now known to be caused by mutations in mismatch repair genes, with different distinct gene mutation patterns explaining the variability in phenotype [3]. Such errors are particularly prominent in microsatellites where the DNA sequence is repetitive and are said to demonstrate instability. It is this area that there is much interest currently to account for young patients presenting with cancers of the colon.

Regular screening of young individuals for colorectal cancer with positive risk factors with regular colonoscopy and genetic testing for FAP and HNPCC (testing for microsatellite instability)[4] is of great importance and this accounts for the majority of young patients presenting with colorectal carcinoma. In this case, whilst genetic screening proved to be negative, a genetic basis for colorectal carcinoma is still the most likely, but this would probably represent a new mutation.

Patients who develop metastatic arthritis secondary to solid tumours are rare. It is even more uncommon for this to be a presenting symptom.

A review of the English language literature reveals that there have been 22 reported cases of malignant joint effusion associated with solid organ malignancy [5-7]. Although there are a number of malignancies which classically spread to bone, over half of the reported cases with metastatic carcinomatous arthritis were associated with lung cancer. The most commonly affected joint is the knee and symptoms usually present in the joint some time after the diagnosis of the primary cancer has been made.

There have only been three cases reported associated with colon cancer [6-8] and the average age of these patients was 72 years (the individual ages were 62, 73 and 83).

The mechanism by which the primary cancer metastasizes to the synovium has been hypothesised to occur by one of two mechanisms. The first is direct haematogenous spread to the synovium [8]. The second that the carcinoma first metastasizes to the bone and then further spreads to the synovium [9] – this theory would be supported by the bone scan results in our case.

With a high index of clinical suspicion a diagnosis should be sought by examination of the synovial fluid [10], however this is only positive in approximately 50% of cases[12]. If negative, a synovial biopsy is indicated, this reaches a diagnosis in the majority of patients. In 7 reported cases both investigations were negative and a positive diagnosis was only reached at autopsy [11].

Treatment is aimed at symptomatic relief. Radiation therapy has been shown to provide some temporary relief [12], with analgesics being the main form of treatment.

Patients with articular metastasis have a worse prognosis, with an average survival of less than 5 months from the time of diagnosis [13].

## Conclusion

The metastatic spread of cancer to the joint and the synovium is one of the rarest manifestations of malignant disease and has not been previously reported as the presenting symptom of disease. The diagnosis is a difficult one to reach and is associated with a poor prognosis. This case illustrates the importance of thorough investigation in reaching this diagnosis and entertaining the possibility in individuals who do not respond to conventional management of acute monoarthritis, even in young patients and individuals who do not display any other symptoms of disease.

## Conflict of interest

The author(s) declare that they have no competing interests.

## Authors' contributions

**NR **and HA conceived the report

**NR **performed the literature search and drafted the manuscript and multiple revisions.

**CC **advised and revised the initial manuscript.

All authors read and approved the final manuscript.
